# Supplementary material for: The Small RNA Universe of Capitella teleta
Source: Front Mol Biosci. 2022 Feb 25;9:802814. doi: 10.3389/fmolb.2022.802814 (PMC8915122; doi:10.3389/fmolb.2022.802814)
Supplement: Supplementary file 1 [file DataSheet1.ZIP › Supplement/known/CAPTEscaffold_192_13671.pdf]

Provisional ID	:	CAPTEscaffold_192_13671
Score total	:	269911.3
Score for star read(s)	:	3.9
Score for read counts	:	269901.5
Score for mfe	:	1.3
Score for randfold	:	1.6
Score for cons. seed	:	3
Total read count	:	529412
Mature read count	:	497907
Loop read count	:	0
Star read count	:	31505

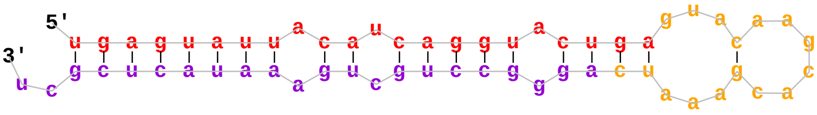

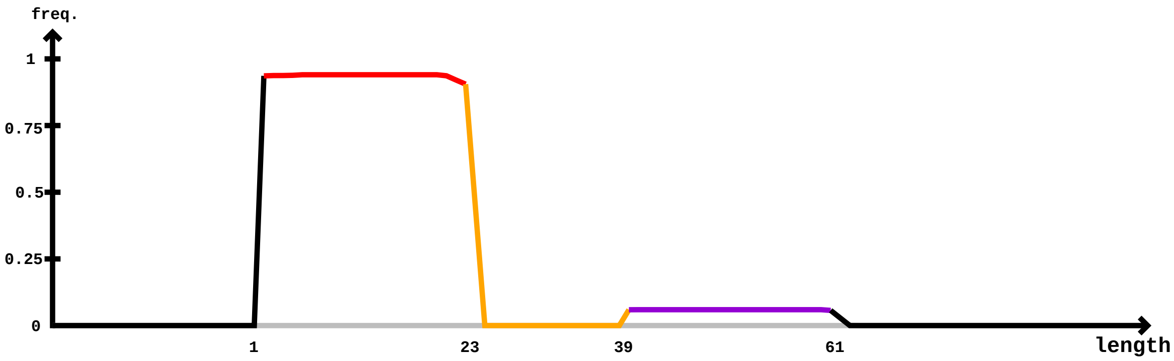

Mature Star

```
5'- uggaaguggcggucagagccugaguauuacaucagguacugaguacaagcacgaaaucagggccugcugaaauacucgcucucugucagcacuaaucaacuaacaagucuaa  -3'  obs
    uggaaguggcggucagagccugaguauuacaucagguacugaguacaagcacgaaaucagggccugcugaaauacucgcucucugucagcacuaaucaacuaacaagucuaa       exp
    uggaaguggcggucagagccugaguauuacaucagguacugaguacaagcacgaaaucagggccugcugaaauacucgcucucugucagcacuaaucaacuaacaagucuaa       known
    ....((((..((.(((((..((((((((.((.(((((.((((...(......)...)))).))))).)).))))))))..)))))))..))))...................   reads   mm   sample

    uggaaguggcggucagagcc............................................................................................   1       0    seq
    ..................Ucugaguauuacaucagguacug.......................................................................   3       1    seq
    ...................cugaguauuacaucagguacuga......................................................................   4       0    seq
    ....................ugaguGuuacaucagguac.........................................................................   1       1    seq
    ....................ugaguauuacaAcagguac.........................................................................   1       1    seq
    ....................ugaguauuacaucagguaA.........................................................................   1       1    seq
    ....................ugaguauuacaucagguac.........................................................................   1946    0    seq
    ....................ugaguauuacaucagguUc.........................................................................   1       1    seq
    ....................ugaguauuacaCcagguac.........................................................................   1       1    seq
    ....................ugaguauuacGucagguac.........................................................................   1       1    seq
    ....................ugaguauuacauUagguac.........................................................................   1       1    seq
    ....................Agaguauuacaucagguac.........................................................................   5       1    seq
    ....................ugaguauuGcaucagguac.........................................................................   4       1    seq
    ....................ugaguauuacaucagguGc.........................................................................   1       1    seq
    ....................ugaguauuacaucaggAac.........................................................................   3       1    seq
    ....................Ggaguauuacaucagguac.........................................................................   1       1    seq
    ....................ugaguauuUcaucagguac.........................................................................   1       1    seq
    ....................ugaguauuacaucGgguac.........................................................................   2       1    seq
    ....................uAaguauuacaucagguac.........................................................................   3       1    seq
    ....................ugGguauuacaucagguac.........................................................................   1       1    seq
    ....................ugaguauuacauAagguac.........................................................................   2       1    seq
    ....................ugaguauuacGucagguacu........................................................................   1       1    seq
    ....................ugaguauuUcaucagguacu........................................................................   2       1    seq
    ....................ugaCuauuacaucagguacu........................................................................   1       1    seq
    ....................ugagAauuacaucagguacu........................................................................   1       1    seq
    ....................ugaguUuuacaucagguacu........................................................................   1       1    seq
    ....................ugaguauuacaucagguaAu........................................................................   1       1    seq
    ....................ugaguauuacauUagguacu........................................................................   3       1    seq
    ....................ugaguauAacaucagguacu........................................................................   3       1    seq
    ....................ugaguCuuacaucagguacu........................................................................   1       1    seq
    ....................ugaguauuacaucaggGacu........................................................................   1       1    seq
```

Mature **Star**

```
uggaaguggcggucagagccugaguauuacaucagguacugaguacaagcacgaaaucagggccugcugaaauacucgcucucugucagcacuaaucaacuaacaagucuaa
....................ugaguauuacauAagguacu........................................................................ 3 1 seq
....................Agaguauuacaucagguacu........................................................................ 24 1 seq
....................ugaguauuaAaucagguacu........................................................................ 4 1 seq
....................ugagCauuacaucagguacu........................................................................ 1 1 seq
....................ugaguauuaUaucagguacu........................................................................ 2 1 seq
....................uAaguauuacaucagguacu........................................................................ 1 1 seq
....................ugaguauuacaucaAguacu........................................................................ 2 1 seq
....................ugaguauuacaucaUguacu........................................................................ 1 1 seq
....................uUaguauuacaucagguacu........................................................................ 1 1 seq
....................ugUguauuacaucagguacu........................................................................ 2 1 seq
....................ugaguauuacaucUgguacu........................................................................ 1 1 seq
....................ugaguauuacaucagguGcu........................................................................ 2 1 seq
....................ugaguauuacaucagguacu........................................................................ 8325 0 seq
....................ugaguaGuacaucagguacu........................................................................ 1 1 seq
....................ugaguaCuacaucagguacu........................................................................ 1 1 seq
....................Ggaguauuacaucagguacu........................................................................ 2 1 seq
....................Ngaguauuacaucagguacu........................................................................ 3 1 seq
....................ugaguauuacaucaggAacu........................................................................ 5 1 seq
....................ugaguGuuacaucagguacu........................................................................ 4 1 seq
....................ugGguauuacaucagguacu........................................................................ 1 1 seq
....................ugaguauuGcaucagguacu........................................................................ 48 1 seq
....................ugaguauuacaucaCguacu........................................................................ 1 1 seq
....................ugaguauuacaucagguacC........................................................................ 1 1 seq
....................ugaguauuacaucagguacA........................................................................ 5 1 seq
....................ugagGauuacaucagguacu........................................................................ 1 1 seq
....................ugaguauuacaucGgguacu........................................................................ 1 1 seq
....................ugaguauuacaucagguacG........................................................................ 6 1 seq
....................Cgaguauuacaucagguacu........................................................................ 1 1 seq
....................ugaguaAuacaucagguacu........................................................................ 3 1 seq
....................Ngaguauuacaucagguacug....................................................................... 2 1 seq
....................ugaguaGuacaucagguacug....................................................................... 2 1 seq
....................ugaguauuacaucagguacug....................................................................... 7812 0 seq
....................Ggaguauuacaucagguacug....................................................................... 6 1 seq
....................ugaguauuaUaucagguacug....................................................................... 4 1 seq
....................Agaguauuacaucagguacug....................................................................... 29 1 seq
....................ugGguauuacaucagguacug....................................................................... 1 1 seq
....................ugaguauuacaucagguacCg....................................................................... 1 1 seq
....................ugaguaAuacaucagguacug....................................................................... 1 1 seq
....................Cgaguauuacaucagguacug....................................................................... 4 1 seq
....................ugagAauuacaucagguacug....................................................................... 4 1 seq
....................ugaguauCacaucagguacug....................................................................... 2 1 seq
....................ugaguauAacaucagguacug....................................................................... 3 1 seq
....................ugaguauuacaucagguacuA....................................................................... 15 1 seq
....................ugaguauuacaCcagguacug....................................................................... 2 1 seq
....................ugaguauuacaucCgguacug....................................................................... 1 1 seq
....................ugaguauuacaucagguacuC....................................................................... 1 1 seq
....................ugaguauuacaucUgguacug....................................................................... 2 1 seq
....................ugaguauuacaucagguaAug....................................................................... 1 1 seq
....................ugaguauuacaucagguacGg....................................................................... 7 1 seq
....................ugaguGuuacaucagguacug....................................................................... 2 1 seq
....................ugaguauuacaucagguGcug....................................................................... 210 1 seq
....................ugaguauuacUucagguacug....................................................................... 1 1 seq
....................uNaguauuacaucagguacug....................................................................... 1 1 seq
....................ugaguauuacaucaggAacug....................................................................... 1 1 seq
....................ugaUuauuacaucagguacug....................................................................... 2 1 seq
....................uUaguauuacaucagguacug....................................................................... 1 1 seq
....................ugagCauuacaucagguacug....................................................................... 1 1 seq
....................ugaguauuacGucagguacug....................................................................... 2 1 seq
....................ugaguauuacaucaggCacug....................................................................... 5 1 seq
....................ugaguauuacaucagguaUug....................................................................... 2 1 seq
....................uAaguauuacaucagguacug....................................................................... 10 1 seq
....................ugaguauuacaucagguacAg....................................................................... 1 1 seq
....................ugaguauuUcaucagguacug....................................................................... 2 1 seq
....................ugaguauuacauUagguacug....................................................................... 3 1 seq
....................ugUguauuacaucagguacug....................................................................... 1 1 seq
....................ugaguauuaAaucagguacug....................................................................... 5 1 seq
....................ugaguauuacaucaAguacug....................................................................... 5 1 seq
....................ugaguauuGcaucagguacug....................................................................... 16 1 seq
....................ugaguauuacauGagguacug....................................................................... 2 1 seq
....................ugaguUuuacaucagguacug....................................................................... 1 1 seq
```

```
                    Mature                Star
uggaaguggcggucagagccugaguauuacaucagguacugaguacaagcacgaaaucagggccugcugaaauacucgcucucugucagcacuaaucaacuaacaagucuaa
....................ugaguauuacaucagCuacug.......................................................................	1	1	seq
....................uCaguauuacaucagguacug.......................................................................	2	1	seq
....................ugaguauuacauGagguacuga......................................................................	23	1	seq
....................ugaguauuacauUagguacuga......................................................................	147	1	seq
....................ugaguauuaNaucagguacuga......................................................................	5	1	seq
....................ugaguauuacaucaCguacuga......................................................................	8	1	seq
....................ugaguauuacaucagguacugN......................................................................	1	1	seq
....................ugaguauAacaucagguacuga......................................................................	105	1	seq
....................ugCguauuacaucagguacuga......................................................................	5	1	seq
....................ugaCuauuacaucagguacuga......................................................................	4	1	seq
....................ugaguauuacaGcagguacuga......................................................................	32	1	seq
....................ugaguauuacaucUgguacuga......................................................................	40	1	seq
....................ugaguCuuacaucagguacuga......................................................................	4	1	seq
....................ugaguauuacaucagguacuga......................................................................	447606	0	seq
....................ugaguNuuacaucagguacuga......................................................................	7	1	seq
....................ugaguauuacaucaggGacuga......................................................................	33	1	seq
....................ugaguauGacaucagguacuga......................................................................	2	1	seq
....................ugaguauuacaucCgguacuga......................................................................	17	1	seq
....................uCaguauuacaucagguacuga......................................................................	37	1	seq
....................ugaguauuacaucagguaNuga......................................................................	1	1	seq
....................ugaguauuacaucagguacuAa......................................................................	277	1	seq
....................ugaguauuacaucagCuacuga......................................................................	22	1	seq
....................ugaguauuacaucagguaUuga......................................................................	26	1	seq
....................ugaguaAuacaucagguacuga......................................................................	168	1	seq
....................Ngaguauuacaucagguacuga......................................................................	157	1	seq
....................ugaguauuacCucagguacuga......................................................................	4	1	seq
....................ugaguauuacaucagguacugG......................................................................	1902	1	seq
....................Ggaguauuacaucagguacuga......................................................................	177	1	seq
....................ugagCauuacaucagguacuga......................................................................	38	1	seq
....................ugaguauuacaucagguCcuga......................................................................	7	1	seq
....................ugaguauuacauAagguacuga......................................................................	171	1	seq
....................ugaguauuacaucagguacuCa......................................................................	35	1	seq
....................ugaguauuacaCcagguacuga......................................................................	41	1	seq
....................ugaguauuacaucagguUcuga......................................................................	48	1	seq
....................ugaguauuacaucagUuacuga......................................................................	12	1	seq
....................ugaguauuacaucagguacGga......................................................................	27	1	seq
....................ugaguauCacaucagguacuga......................................................................	56	1	seq
....................ugaguauuacaucagguacugU......................................................................	2877	1	seq
....................Agaguauuacaucagguacuga......................................................................	1337	1	seq
....................ugaguauuacaucagguacNga......................................................................	1	1	seq
....................ugaguauuacUucagguacuga......................................................................	44	1	seq
....................ugaguauuacaucagguacuUa......................................................................	28	1	seq
....................ugaguauuacGucagguacuga......................................................................	76	1	seq
....................ugagGauuacaucagguacuga......................................................................	25	1	seq
....................ugaguaGuacaucagguacuga......................................................................	31	1	seq
....................ugaguauuacaucagguaGuga......................................................................	24	1	seq
....................ugaguauuaGaucagguacuga......................................................................	9	1	seq
....................ugaguauuCcaucagguacuga......................................................................	8	1	seq
....................Cgaguauuacaucagguacuga......................................................................	86	1	seq
....................ugaguGuuacaucagguacuga......................................................................	1286	1	seq
....................ugaguauuacaucagguaAuga......................................................................	64	1	seq
....................ugaguauuaUaucagguacuga......................................................................	135	1	seq
....................ugaguauuacaucaAguacuga......................................................................	115	1	seq
....................ugaguauuacaucGgguacuga......................................................................	83	1	seq
....................ugagAauuacaucagguacuga......................................................................	296	1	seq
....................ugaUuauuacaucagguacuga......................................................................	115	1	seq
....................ugaguUuuacaucagguacuga......................................................................	38	1	seq
....................ugaguauuacaAcagguacuga......................................................................	80	1	seq
....................uUaguauuacaucagguacuga......................................................................	70	1	seq
....................uAaguauuacaucagguacuga......................................................................	417	1	seq
....................ugaguauUcaucagguacuga.......................................................................	68	1	seq
....................ugGguauuacaucagguacuga......................................................................	353	1	seq
....................ugNguauuacaucagguacuga......................................................................	5	1	seq
....................ugaguauuacaucagguacCga......................................................................	57	1	seq
....................ugaguaCuacaucagguacuga......................................................................	52	1	seq
....................ugaguauuacaucaggCacuga......................................................................	49	1	seq
....................ugaguauuacaucagguacugC......................................................................	23	1	seq
....................ugaguauuacaucaggAacuga......................................................................	111	1	seq
....................ugaAuauuacaucagguacuga......................................................................	44	1	seq
....................ugaguauuacaucaUguacuga......................................................................	18	1	seq
```

Mature **Star**

```
uggaaguggcggucagagccugaguauuacaucagguacugaguacaagcacgaaaucagggccugcugaaauacucgcucucugucagcacuaaucaacuaacaagucuaa
....................ugUguauuacaucagguacuga......................................................................    38      1    seq
....................ugaguauuaAaucagguacuga......................................................................    87      1    seq
....................ugaguauuacaucagAuacuga......................................................................    32      1    seq
....................ugaguauuGcaucagguacuga......................................................................    4051    1    seq
....................uNaguauuacaucagguacuga......................................................................    20      1    seq
....................ugaguauuacaucagguacAga......................................................................    95      1    seq
....................ugaguauuacaucagguGcuga......................................................................    63      1    seq
....................ugaguauuacaucagguacugaA.....................................................................    1469    1    seq
....................ugaguauuacaucagguacugag.....................................................................    21      0    seq
....................ugaguauuacaucagguacugaU.....................................................................    11871   1    seq
....................ugaguauuacaucagguacugaC.....................................................................    5       1    seq
....................ugaguauuacaucagguacugaUu....................................................................    23      1    seq
....................ugaguauuacaucagguacugagu....................................................................    2       0    seq
....................ugaguauuacaucagguacugaAu....................................................................    15      1    seq
.....................gaguauuacaucagguac.........................................................................    1       0    seq
.....................gaguauuacaucagguacu........................................................................    1       0    seq
.....................Uaguauuacaucagguacug.......................................................................    1       1    seq
.....................gaguauuacaucagguacug.......................................................................    25      0    seq
.....................gaguauuacaGcagguacuga......................................................................    1       1    seq
.....................gaguauuacaucagguacugU......................................................................    2       1    seq
.....................gaguauuGcaucagguacuga......................................................................    7       1    seq
.....................gaguauuacaucagguacugG......................................................................    1       1    seq
.....................gaguauuacaucagguacuga......................................................................    509     0    seq
.....................gaguauuacaucagguacGga......................................................................    1       1    seq
.....................gaguauuacaAcagguacuga......................................................................    6       1    seq
.....................gaguauuacauAagguacuga......................................................................    36      1    seq
.....................gaguauuacaucagguUcuga......................................................................    2       1    seq
.....................gaguauuacaucagguaAuga......................................................................    1       1    seq
.....................Uaguauuacaucagguacuga......................................................................    6       1    seq
.....................gaguauuacaucagguacAga......................................................................    1       1    seq
.....................gaguauuUcaucagguacuga......................................................................    4       1    seq
.....................gaguGuuacaucagguacuga......................................................................    1       1    seq
.....................gaguauuacaucagguacugaU.....................................................................    16      1    seq
......................Uguauuacaucagguacuga......................................................................    1       1    seq
......................aguauuacaucagguacuga......................................................................    69      0    seq
.......................guauuacaucagguacugU......................................................................    1       1    seq
.......................guauuacaucGgguacuga......................................................................    1       1    seq
.......................Auauuacaucagguacuga......................................................................    1       1    seq
.......................guauuacaucagguacuga......................................................................    430     0    seq
.......................guauuacauAagguacuga......................................................................    1       1    seq
.......................guauuacaucagguacugG......................................................................    2       1    seq
.......................guauuaUaucagguacuga......................................................................    1       1    seq
........................Nauuacaucagguacuga......................................................................    1       1    seq
........................uauuacaucagguUcuga......................................................................    1       1    seq
........................uGuuacaucagguacuga......................................................................    1       1    seq
........................uauuacaucagguacuga......................................................................    1064    0    seq
........................uaAuacaucagguacuga......................................................................    2       1    seq
........................uaCuacaucagguacuga......................................................................    1       1    seq
........................uauuacaucagguacugG......................................................................    4       1    seq
........................uauuacaucagCuacuga......................................................................    1       1    seq
........................uauuGcaucagguacuga......................................................................    9       1    seq
........................Aauuacaucagguacuga......................................................................    2       1    seq
........................uauuacauUagguacuga......................................................................    1       1    seq
.........................auuacaucagguacugaU.....................................................................    1       1    seq
..........................................guacaagcacgaaaucagggccugc.............................................    1       0    seq
........................................................ucagggccugcugaaauacucgc.................................    4       0    seq
.........................................................cagggccugcugaaauacucgcu................................    2       0    seq
..........................................................Ggggccugcugaaauacu....................................    1       1    seq
..........................................................agggccugcugaaauacu....................................    12      0    seq
..........................................................agAgccugcugaaauacu....................................    1       1    seq
..........................................................agggccugcugaaauacucg..................................    6       0    seq
..........................................................agggccugcugaaauacucU..................................    4       1    seq
..........................................................agggccugcugaaauacuAgc.................................    1       1    seq
..........................................................agggccugcuAaaauacucgc.................................    1       1    seq
..........................................................Ggggccugcugaaauacucgc.................................    7       1    seq
..........................................................agCgccugcugaaauacucgc.................................    1       1    seq
..........................................................agggccugcugaaauacuUgc.................................    1       1    seq
..........................................................Ngggccugcugaaauacucgc.................................    2       1    seq
..........................................................agggccugcugGaauacucgc.................................    1       1    seq
..........................................................agggccugcugaaauacucgc.................................    1354    0    seq
```

```
                    Mature                                      Star
uggaaguggcggucagagccugaguauuacaucagguacugaguacaagcacgaaaucagggccugcugaaauacucgcucucugucagcacuaaucaacuaacaagucuaa
..........................................................agggccuCcugaaauacucgc.................................   1       1       seq
..........................................................agggccugcugaGauacucgcu................................   6       1       seq
..........................................................agggccugcugaaauaAucgcu................................   3       1       seq
..........................................................agggccugcugaaUuacucgcu................................   4       1       seq
..........................................................agggccugcugaaauacucAcu................................   7       1       seq
..........................................................agggccugcugaaauacucgcC................................   5       1       seq
..........................................................agggccugcugaCauacucgcu................................   1       1       seq
..........................................................Ggggccugcugaaauacucgcu................................   310     1       seq
..........................................................aUggccugcugaaauacucgcu................................   5       1       seq
..........................................................agggccugcugaaauacucgcu................................   29127   0       seq
..........................................................agUgccugcugaaauacucgcu................................   4       1       seq
..........................................................agggccugcugaaaGacucgcu................................   2       1       seq
..........................................................agggccugcugUaauacucgcu................................   4       1       seq
..........................................................agggGcugcugaaauacucgcu................................   2       1       seq
..........................................................agggccugcGgaaauacucgcu................................   3       1       seq
..........................................................agggccugcuUaaauacucgcu................................   3       1       seq
..........................................................agggccugcugaaauacuGgcu................................   6       1       seq
..........................................................agggccugcugaaauGcucgcu................................   3       1       seq
..........................................................agggcNugcugaaauacucgcu................................   2       1       seq
..........................................................agggccuUcugaaauacucgcu................................   3       1       seq
..........................................................agggcGugcugaaauacucgcu................................   1       1       seq
..........................................................agggccuAcugaaauacucgcu................................   8       1       seq
..........................................................agggccugcugGaauacucgcu................................   7       1       seq
..........................................................agggccugcugaaauacucgcA................................   38      1       seq
..........................................................agCgccugcugaaauacucgcu................................   8       1       seq
..........................................................agggccAgcugaaauacucgcu................................   26      1       seq
..........................................................agggccugcugaaauaUucgcu................................   1       1       seq
..........................................................agggccugAugaaauacucgcu................................   8       1       seq
..........................................................agAgccugcugaaauacucgcu................................   14      1       seq
..........................................................agggccugcugaaauacucgcG................................   47      1       seq
..........................................................agggccugcugaaauacuUgcu................................   16      1       seq
..........................................................agggccugcugaaauacGcgcu................................   2       1       seq
..........................................................agggccugcugaaauacAcgcu................................   11      1       seq
..........................................................agggcUugcugaaauacucgcu................................   2       1       seq
..........................................................agggAcugcugaaauacucgcu................................   5       1       seq
..........................................................agggccugcugaaauacucUcu................................   6       1       seq
..........................................................agggccugcugaaauacucCcu................................   3       1       seq
..........................................................Ugggccugcugaaauacucgcu................................   1       1       seq
..........................................................agggccugcugaaGuacucgcu................................   6       1       seq
..........................................................agggccugcugaaaAacucgcu................................   5       1       seq
..........................................................agggccugcugaaauacucgAu................................   10      1       seq
..........................................................Cgggccugcugaaauacucgcu................................   12      1       seq
..........................................................Ngggccugcugaaauacucgcu................................   3       1       seq
..........................................................agggccugcugaaauaGucgcu................................   1       1       seq
..........................................................agggccugcugaaauacuAgcu................................   13      1       seq
..........................................................agggcAugcugaaauacucgcu................................   9       1       seq
..........................................................agggccCgcugaaauacucgcu................................   4       1       seq
..........................................................agggccugcAgaaauacucgcu................................   21      1       seq
..........................................................agggccGgcugaaauacucgcu................................   2       1       seq
..........................................................agggccugcuCaaauacucgcu................................   4       1       seq
..........................................................agggccugcugaaauUcucgcu................................   3       1       seq
..........................................................agggccugcugaUauacucgcu................................   3       1       seq
..........................................................aggUccugcugaaauacucgcu................................   2       1       seq
..........................................................aggAccugcugaaauacucgcu................................   3       1       seq
..........................................................agggccugcugaaaCacucgcu................................   3       1       seq
..........................................................agggccugcugaaauacCcgcu................................   2       1       seq
..........................................................agggUcugcugaaauacucgcu................................   3       1       seq
..........................................................agggccuCcugaaauacucgcu................................   1       1       seq
..........................................................agggccugGugaaauacucgcu................................   2       1       seq
..........................................................aAggccugcugaaauacucgcu................................   37      1       seq
..........................................................agggccugcCgaaauacucgcu................................   12      1       seq
..........................................................aCggccugcugaaauacucgcu................................   1       1       seq
..........................................................agggccugcuAaaauacucgcu................................   102     1       seq
..........................................................agggccugcugaaauacucgcuU...............................   12      1       seq
..........................................................agggccugcugaaauacucgcuA...............................   2       1       seq
..........................................................agggccugcugaaauacucgcuAu..............................   1       1       seq
..........................................................agggccugcugaaauacucgcuUu..............................   22      1       seq
...........................................................gggccugcugaaauacucgc.................................   1       0       seq
...........................................................gggGcugcugaaauacucgcu................................   2       1       seq
...........................................................gggccugcugaaauacucgcu................................   90      0       seq
```

Mature Star

```
uggaaguggcggucagagccugaguauuacaucagguacugaguacaagcacgaaaucagggccugcugaaauacucgcucucugucagcacuaaucaacuaacaagucuaa
...........................................................Aggccugcugaaauacucgcu................................   2   1   seq
...........................................................gggccAgcugaaauacucgcu................................   2   1   seq
............................................................ggccugcugaaauacucgc.................................   1   0   seq
............................................................ggccugcugaaauacucgcu................................   1   0   seq
..............................................................ccugcugaaauacucgcu................................   2   0   seq
................................................................................cucugucagcacuaaucaac............   2   0   seq
................................................................................cucugucagcacuaaucaacu...........   1   0   seq
................................................................................cucugucagcacuaaucaacuaacaagu....   3   0   seq
```
